# Nitrosative stress in human skeletal muscle attenuated by exercise countermeasure after chronic disuse^☆^

**DOI:** 10.1016/j.redox.2013.10.006

**Published:** 2013-10-28

**Authors:** Michele Salanova, Gudrun Schiffl, Martina Gutsmann, Dieter Felsenberg, Sandra Furlan, Pompeo Volpe, Andrew Clarke, Dieter Blottner

**Affiliations:** a Charité - Universitätsmedizin Berlin, Center for Space Medicine Berlin (ZWMB) Germany; b Charité - Universitätsmedizin Berlin, Department of Anatomy, Neuromuscular Group, Berlin, Germany; c Charité - Universitätsmedizin Berlin, Center of Muscle and Bone Research (ZMK) Berlin, Germany; d Dipartimento di Scienze Biomediche, Università di Padova, Italy; C.N.R. Institute of Neuroscience, Padova, Italy

**Keywords:** Nitric oxide synthase, Calcium-release channels, Calcium ATPase, Nitrosative stress, Nrf-2, Skeletal muscle, Calcium homeostasis

## Abstract

Activity-induced nitric oxide (NO) imbalance and “nitrosative stress” are proposed mechanisms of disrupted Ca^2+^ homeostasis in atrophic skeletal muscle. We thus mapped S-nitrosylated (SNO) functional muscle proteins in healthy male subjects in a long-term bed rest study (BBR2-2 Study) without and with exercise as countermeasure in order to assess (i) the negative effects of chronic muscle disuse by nitrosative stress, (ii) to test for possible attenuation by exercise countermeasure in bed rest and (iii) to identify new NO target proteins. Muscle biopsies from calf soleus and hip vastus lateralis were harvested at start (Pre) and at end (End) from a bed rest disuse control group (CTR, *n*=9) and two bed rest resistive exercise groups either without (RE, *n*=7) or with superimposed vibration stimuli (RVE, *n*=7). At subcellular compartments, strong anti-SNO-Cys immunofluorescence patterns in control muscle fibers after bed rest returned to baseline following vibration exercise. Total SNO-protein levels, Nrf-2 gene expression and nucleocytoplasmic shuttling were changed to varying degrees in all groups. Excess SNO-protein levels of specific calcium release/uptake proteins (SNO-RyR1, –SERCA1 and –PMCA) and of contractile myosin heavy chains seen in biopsy samples of chronically disused skeletal muscle were largely reduced by vibration exercise. We also identified NOS1 as a novel NO target in human skeletal muscle controlled by activity driven auto-nitrosylation mechanisms. Our findings suggest that aberrant levels of functional SNO-proteins represent signatures of uncontrolled nitrosative stress management in disused human skeletal muscle that can be offset by exercise as countermeasure.

## Highlights

• NOS1 is auto-S-nitrosylated (SNO-NOS1) in human skeletal muscle.
• Excess levels of functional SNO-proteins in disused-induced muscle atrophy.
• Recovered levels of functional SNO-proteins by exercise countermeasure.
• Aberrant levels of SNO-proteins are signatures of nitrosative stress management.
• Vibration intervention induced anti-oxidative master gene Nrf2 myonuclear shuttling.

## Graphical abstract

**Figure:**
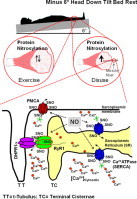

## Introduction

S-nitrosylation is a posttranslational and reversible redox-based modification of functional proteins and a major source of nitric oxide (NO) bioactivity thus regulating cellular dynamics and plasticity in a wide variety of tissues and cell types [1]. By contrast, uncontrolled over-production or aberrant levels of free radicals such as superoxide (O_2_^−^) or nitric oxide (NO) in cells and tissues provide the basis for oxidative/nitrosative stress induced by “reactive oxygen/nitrogen species” (ROS/RNS) that increasingly play central roles in numerous disease states including various myopathies [2]. For instance, skeletal muscle atrophy induced by prolonged disuse may induce oxidative stress mechanisms that accelerate myofiber protein degradation [3] via increased substrate recognition of oxidized proteins by E3 ligases (MuRF1-3) which in turn, promote their susceptibility to proteolysis [4].

An essential step in nitrosative stress is the covalent modification of a reactive cysteine residue (–SH) by nitric oxide (NO), termed *S*-nitrosation or *S*-nitrosylation (–SH groups are converted into –SNO groups) in a given target protein [1]. The reversible regulation of SNO-protein functions led to the view that nitrosothiols most likely act as posttranslational modifications analogous to phosphorylation or acetylation of functional target proteins [1]. Nitrosothiols are however exceptionally labile because of their highly reactive potential for intracellular physiological reducing agents in vivo, such as ascorbic acid and reduced glutathione (GSH) [5], or reduced metal ions [6]. SNO-protein modifications may have a tissue half-life of a few seconds to a few minutes maximum [5] thus making them particularly difficult to study biochemically.

Recently, a biochemical assay has been developed to study SNO-proteins by their labeling with a stable biotin linker specifically bound to *S*-nitrosylated cysteines [7]. The “biotin-switch technique” (BST) should therefore provide the basis for indirect SNO-protein detection and purification of muscular proteins as putative SNO targets via endogenous nitric oxide (NO) produced by NO-synthases (NOSs) known to be moderately expressed in normal human skeletal muscle [8,9] and, for example, down- or upregulated in atrophic vs. active muscle following extended bed rest [10]. Vertebrate skeletal muscle is a major source of NO signals [8] that are key regulators in skeletal muscle biology including open/close probability of calcium release channels [11]. The consequences of long-lasting nitrosative stress conditions following muscle disuse/inactivity in long-term bed rest and its attenuation by exercise countermeasure during bed rest remains an open issue.

Given the eminent role of Ca^2+^ homeostasis in skeletal muscle physiology, and the fact that dysregulation of intermediate signaling processes underlying Ca^2+^ homeostasis are closely linked to disuse-induced muscle atrophy [12,13], we investigated whether altered levels of subcellular functional SNO-proteins from sarcolemmal and sarcoplasmic reticulum calcium-release and -uptake proteins of a slow or a fast type human leg muscle (*soleus*, *vastus lateralis*) were detectable in muscle biopsies before and after 60 days of 6° head down tilt (HDT) bed rest, i.e., an experimental immobilisation model that makes use of lower body unloading resulting in leg muscle disuse, thus permitting the study of neuromusculoskeletal system deconditioning in healthy subjects [14]. The bed rest study protocol consisted of two different exercise plus one control group [15]. This allowed us to address the ultimate question whether muscle activity on its own would be able to significantly reduce muscular SNO-protein dysregulation, thus mitigating nitrosative stress as a possible negative regulator of atrophy in human leg skeletal muscles following long-term bed rest. Further, we might be able to assess which of the two different exercise protocols, resistive-only (RE) or resistive vibration exercise (RVE), might be able to more effectively prevent maladaptation in disused skeletal muscle.

Prolonged muscle disuse in long-term bed rest significantly increased the levels of functional SNO-muscle proteins involved in Ca^2+−^homeostasis of a mixed fast-type *vastus lateralis* or slow-type *soleus* in a muscle-specific way. We also provide evidence for auto-S-nitrosylation of NOS1 proteins, a major source of NO in human skeletal muscle fibers [6], and a new candidate on the list of functional SNO-proteins. Some of these changes were significantly offset by RVE, and to lesser extent, by RE countermeasure in bed rest.

## Materials and methods

### Bedrest study protocol and biopsies

The 2nd Berlin BedRest Study (BBR2-2) was conducted in the years 2007–08 at the Charité Campus Benjamin Franklin in Berlin, Germany. The detailed protocol of the randomized controlled BBR2-2 study including description of the two exercise countermeasure trials (RE, RVE) is published elsewhere [15]. Briefly, twenty-three healthy male subjects underwent 60 days of head-down tilt (HDT) bed rest. Subjects were randomized to an inactive bed rest control group (CTR, mean age 33.1±7.8 yr; *n*=9), a bed rest high-load resistive exercise group (RE, mean age 31.1±5.1 yr; *n*=7), and a bed rest group that combined high-load resistive exercise with whole-body vibration (RVE, mean age 32.2±10.4 yr; *n*=7). Exercise (RE or RVE) was performed on the same custom made Galileo Space trainer (Novotec Medical GmbH, Pforzheim, Germany) while in HDT position, whereas CTR subjects remained strictly in the HDT supine position without being trained during the bed rest campaign.

In all groups, open muscle biopsies from *soleus* (SOL) and *vastus lateralis* (VL) muscles were performed six days before (BDC-6) start (Pre) and two days before (HDT58) the end (End) of bed rest [15]. The Charité Universitätsmedizin Berlin Ethics Committee approved this study in accordance to the World Medical Association Code of Ethics, Declaration of Helsinki (1964). Subjects gave their informed written consent prior to participation in the study and were aware that they were permitted to withdraw from the study at any time.

### Reagents

For the detection of nitrosylated proteins we used the S-Nitrosylated Protein Detection Kit Cayman (Cayman Chemical Item Number 10006518). The high capacity streptavidin agarose resin (PN20361) from Thermo Scientific was employed (Thermo Fisher Scientific, Rockford, USA). Monoclonal antibodies specific for the slow myosin heavy chain (sMyHC) (mouse IgG1, clone NOQ7.5.4D), the fast myosin heavy chain isoforms (fMyHC) (clone My-32), the *α*-tubulin (mouse IgG1, clone DM19), and polyclonal (rabbit) for SNO-Cys (N5411) from Sigma (St. Louis, MO, USA) were used. Monoclonal antibody specific for Dystrophin (mouse IgG1, clone Dy8/6C5) were from Novocastra Laboratories (Newcastle on Tyne, UK). Monoclonal antibodies for NOS1 (SC-5302), for TRPC1 (SC-133076) and for nuclear factor-erythroid-2-related factor 2 (Nrf-2) (SC-365949; rabbit SC-13032) were from Santa Cruz Biotechnology (Santa Cruz, CA). Monoclonal antibodies for RyR1 were from Alexis Inc. (mouse IgG1, clone 34C, ALX-804-016-R100). Monoclonal antibodies for DHPR1*α* (Clone 1A, AB2862) and for SERCA1a (clone VE121G9, ab2819) were from Abcam (Abcam Ltd., UK Cambridge). Monoclonal antibodies for PMCA1 (LS-C87407) were from LifeSpan (LifeSpan BioSciences, Inc). Secondary antibodies Alexa 488-conjugated and/or Alexa 555-conjugated affinity-purified goat anti-mouse and anti-rabbit were from Invitrogen (Invitrogen Corporation, CA, USA); alkaline phosphatase-conjugated (AP) secondary antibodies (Dako^®^, Glostrup, Denmark) were visualized with the one step NBT (P-Nitro Blue Tetrazolium chloride)/BCIP (5-Bromo-4-Chloro-3-Indolyl Phosphate) alkaline substrate solution buffer from Pierce (Thermo Fisher Scientific, LSR Rockford, IL, USA). Protease inhibitor cocktail and Tris–HCl were from Boehringer Mannheim (Roche Diagnostics, Mannheim, Germany). All other chemicals were of analytical and molecular grade. All primary antibodies were diluted at final concentration of 2 µg/ml. Secondary AP-conjugated antibodies were diluted 1:500. Fluorochrome-conjugated secondary antibodies were diluted 1:2000 final concentration.

### Biotin switch technique (BST)

Equal amounts of muscle biopsy (10 mg) from each Pre and End bed rest subject (CTR *n*=9; RE *n*=7; RVE *n*=7) were analysed according to BST established previously [7]. Briefly, free reactive thiol groups were first blocked by incubation of samples with the thiol-specific methylthiolating agent methyl methanethiosulfonate (MMTS) in the presence of sodium dodecyl sulfate (SDS) to ensure access of MMTS to buried cysteines. After blocking free thiols, nitrosothiol bonds were selectively reduced by using ascorbate, which results in the reduction of nitrosothiols to thiols. Total proteins were then precipitated with cold acetone in order to remove free or unreacted MMTS. In the third and final step, the newly formed thiols were reacted with *N*-[6-(biotinamido)hexyl]-3′-(2′-pyridyldithio)-propionamide (biotin-HPDP), a sulfhydryl-specific biotinylating reagent thus the previously S-nitrosylated cysteine residues are finally biotinylated. Control experiments were performed by omitting the biotin-HPDP. Analysis of biotinylated muscle proteins was done blinded using coded samples at least by triple determination each protein.

### Dot-blot analysis

Equal amounts (vol. 3 µl) of biotin-labeled proteins from each group were spotted on a nitrocellulose membrane using a “BioRad dot-blot vacuum chamber”, and thereafter incubated in a dry box at 40 °C for 30 min, washed with TBS-T (Tris-Buffered Saline 0.2% Tween^®^ 20), preincubated with blocking buffer (TBS-T 2% BSA) for 30 min., and processed for biotin detection using the alkaline phosphatase-conjugated streptavidin visualized by using the alkaline phosphatase substrate bromochloro-indolylphosphate/nitroblue tetra-zolium salt (BCIP/NBT, Pierce). Proteins were semi-quantified by densitometry analysis (GS-800 device, Quantity-One^™^ software, BioRad Inc., Munich, Germany).

### Streptavidin-agarose affinity chromatography

Biotinylated samples were pooled across the Pre-, respectively End- bed rest subjects of each experimental group to overcome possible limitations in the amount of SNO-proteins or the low relative protein expression in biopsy samples. The biotin-labeled proteins were then separated from the total protein pool by using streptavidin-affinity chromatography covalently bound to agarose beads. Purified biotinylated proteins were then eluted with 10 mM EDTA pH 8.2 and 95% formamide 10 min at high temperature. The presence of SNO-proteins was qualitatively and quantitatively assayed by Western blot analysis followed by densitometry analysis.

### Protein extraction

For Western blot analysis, frozen SOL and VL muscle biopsies were resolved in RIPA buffer (50 mM Tris–HCl ph 7.4, 150 mM NaCl, 1% NP-40, 0.5% sodium deoxicholate, 0.1% SDS, 2 mM MgCl_2_ in presence of a protease inhibitor cocktail). A glass Dounce homogenizer was used with 20 strokes per sample. Tissue extracts were centrifuged at 14,000*g*, 15 min at 4 °C. Protein concentration of the soluble fraction was determined by using a colorimetric assay kit (Pierce) and stock solutions were made and stored at −80 °C until used.

### Western blot analysis

Samples (CTR *n*=9; RE *n*=7; RVE *n*=7) were diluted (to 3 mg/ml final concentration) into an SDS-containing loading buffer, heated to 95 °C for 5 min before an aliquot (10 μL) of each assay sample was loaded onto a Tris–glycine polyacrylamide gel (Bio-Rad). Proteins were resolved by electrophoresis in SDS-containing running buffer. Proteins were transferred to a nitrocellulose membrane and then blocked overnight at 4 °C with TBS-T (Tris-buffered saline, pH 7.5, 0.2% Tween^®^ 20) supplemented with 4% non-fat dry milk. The membrane was probed with the primary antibody in TBS-T supplemented with 4% non-fat dry milk. Rinsed blots were incubated for 1 h with anti-rabbit or anti-mouse or anti-goat alkaline phosphatase-conjugated secondary antibodies diluted 1:500 in TBS–T solution, rinsed again, and finally detected using the alkaline phosphatase substrate bromochloro-indolylphosphate/nitroblue tetrazolium salt (BCIP/NBT, Pierce). Protein content was quantified by densitometry using the GS-800 device (Quantity-One^™^ software, Bio-Rad). α-tubulin was used as a protein load marker.

### SNO-Cys immunohistochemistry and laser confocal analysis

Cryosections (8 µm thickness) from SOL and VL muscles of “Pre” and “End” bed rest groups (*n*=5 each) were double-labeled with commercial antibodies raised against SNO-Cys and NOS1. Primary antibodies were visualized using goat anti-rabbit (Alexa 488-conjugated) and anti-mouse (Alexa 555-conjugated) antibodies respectively diluted at a final concentration of 2 µg/ml. Our protocols included subject-matched (End- vs. Pre-bed rest) cryosections in order to balance inter-subject variability and achieve comparable immunostaining conditions.

#### Confocal microscopy

Immunofluorescence images were acquired with a Leica DM RE7 upright microscope equipped with a three-channel confocal laser system (Leica TCS SP-2, Leica Microsystems, Bensheim, Germany) using the HCX PL APO CS 40x**/**1.25 Oil objective. The relative fluorescence intensity of immunostained subcellular compartments from at least twenty (*n*=20) myofibers from each cyosection of each subject was determined according to an established protocol [10,16]. Briefly, the area pixel intensity of a selected region of interest (ROI) was measured from the digitized confocal image scans and expressed as arbitrary units (a.u.), in the range of 0 to 255 a.u.. Changes of fluorescence intensity referable to SNO-Cys determined in samples of individual subjects and groups, were calculated as the percentage change of a.u. of End- vs. Pre-bed rest (baseline). All digitized images were analyzed using the Leica confocal software (License number: 156108810, Version 2.61.1537).

#### SNO-Cys positive/negative control

Cryosections from human SOL CTR-Pre bed rest were pre-incubated with 50 mM NaNO_2_, 0.5 M HCl, at 37 °C for 20 min. (positive control), or with the blocking reagent HgCl_2_ solution at 0.2% for 30 min. at 37 °C (negative control, according to the A.G. Scientific manufacture protocol), and processed pair-wise for anti-SNO-Cys immunohistochemistry.

### RNA extraction and quantitative real-time polymerase chain reaction (qPCR)

RNA extraction and quantitative Real-Time Polymerase Chain Reaction (qPCR)- RNA was isolated from human skeletal muscles BR biopsies (SOL CTR, RE and RVE *n*=5; VL CTR and RE *n*=5, RVE *n*=4) using RNeasy fibrous tissue mini kit (QIAGEN) according to the manufacturer’s instructions as previously reported by Salanova M. et al. [17]. Briefly, 200 ng of RNA were transcribed in cDNA by using SuperScript^®^ VILO^™^ cDNA Synthesis Kit (Invitrogen). Cyclophilin A (PPIA), beta-2-microglobulin (B2M) and ribosomal protein L32 (RPL32) were used as reference genes [17]. The PCR parameters were initial denaturation at 95 °C for 30 s followed by 40 cycles of 10 s at 95 °C and 30 s at the corresponding annealing temperature (55 °C for Nrf-2 and for PPIA, B2M, and RPL32) for the acquisition of fluorescence signal. All samples were run simultaneously with RNA- and RT-negative controls. Both, a melting curve and normalization were performed as previously reported by Salanova M. et al. [17].

The specific primers for Nrf-2 were the same published by Brocca L. et al. [18]. Specific primers for PPIA, B2M and RPL32 are reported in Salanova M. et al. [17]. Data are expressed as means±SEM. Comparisons were made by using *t*-test, with *P*<0.05 being considered statistically significant.

### Nuclear factor-erythroid-2-related factor 2 (Nrf-2) myonuclei translocation

Human skeletal muscle biopsy cryosections (8 µm thickness), from SOL (*n*=7) and VL (*n*=7) pre- and end-BR groups, were double immunolabelled with anti-Dystrophin and anti-Nrf-2 antibodies. In all Nrf-2 immunostaining protocols we used goat anti-rabbit Alexa-488 for Nrf-2 and goat anti-mouse Alexa-555 for Dystrophin. Nuclei were counterstained with blue DAPI.

Double immunostained muscle cryosections were then inspected by epifluorescence microscopy enabling the detection of Nfr2-accumulation in myonuclei. Particularly, a meandering screening pattern was used for myonuclei detection in both, SOL and VL cryosections. Briefly, more than cytosolic Nrf-2 baseline immunofluorescence Nrf-2 accumulation was defined as Nrf-2 myonuclei translocation positive, whereas a decrease in nuclear Nrf-2 accumulation below cytosolic baseline or cytosol-like nuclear was determined as Nrf-2 myonuclei translocation negative. At least 50 myonuclei were scored each subject/biopsy.

### Statistical analysis

Statistical analyses were performed using SigmaPlot version 9.0 software. Results are given as mean ±SE. Significance of differences of data was analyzed with Student’s *t*-test and by one-way ANOVA. Differences were considered to be significant at *P*≤*0.05* (⁎).

## Results

### Biotinylated proteins reflecting SNO-proteins in human skeletal muscle biopsies following bed rest and exercise countermeasures

**Fig. 1:**
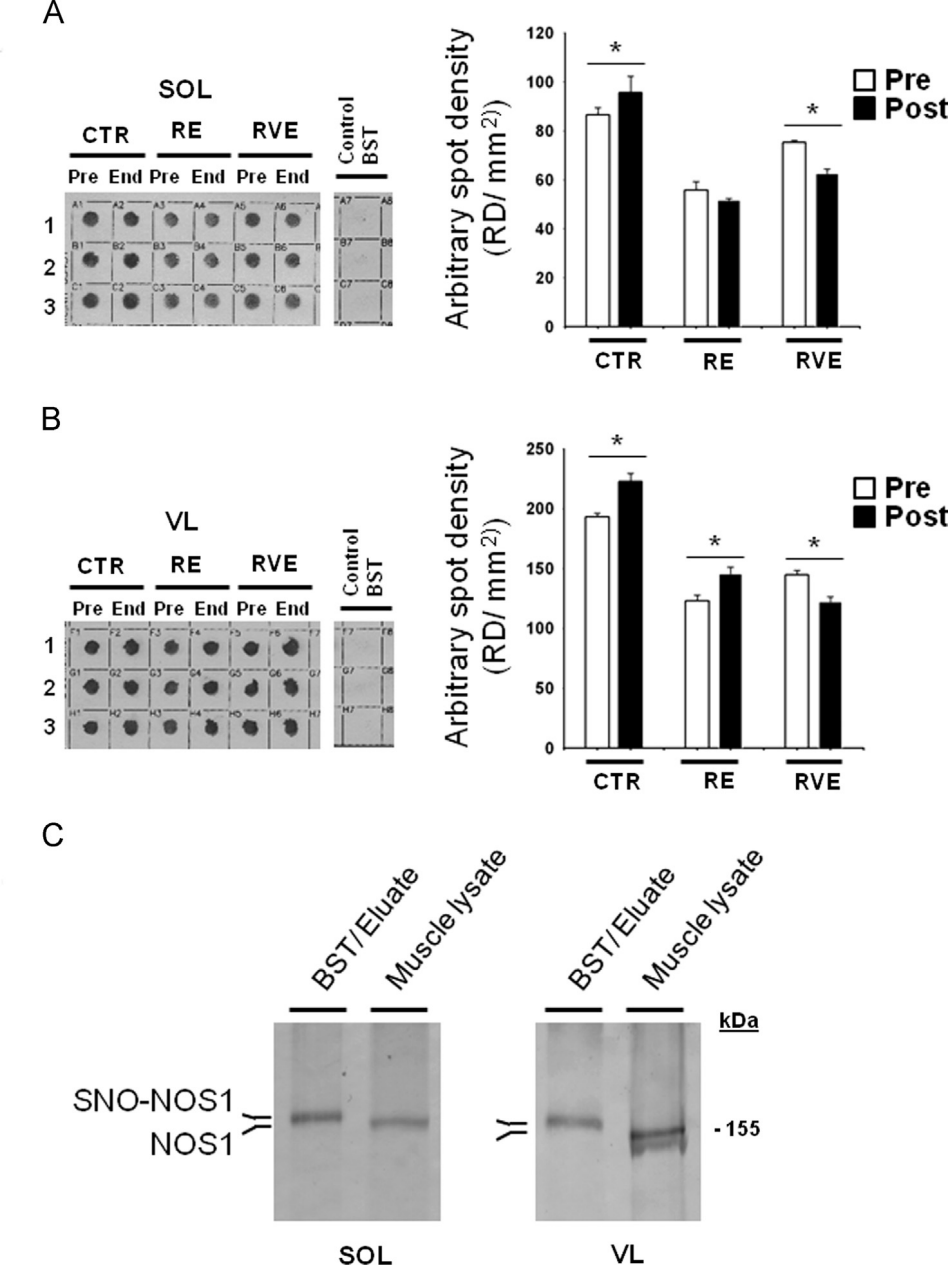
SNO-protein assay in human skeletal muscle biopsies. A. Dot blots analysis (left panel) biotin-labeled proteins (reflecting SNO-proteins) in soleus (SOL) of End vs. Pre bed rest subjects (1,2,3: triplicate), Control BST was obtained by omitting biotin-HPDP during the protocol; right panel, dot blot densitometry analysis. B. (left panel) Dot blot analysis of biotin-labeled proteins in vastus lateralis (VL) of End vs. Pre bed rest subjects (1,2,3: triplicate), Control BST was obtained by omitting biotin-HPDP during the protocol; right panel, dot blot densitometry analysis. A significant increase of biotin incorporation is observed in CTR group (*n*=9) in both muscles (SOL, Pre 86.51±2.9, End 95.64±6.5; VL Pre 192.94±3.07, End 222.59±6.6). In the RE group (*n*=7) a significant increase was in VL only (Pre 123.07±4.7, End 145.6±5.56) but not in SOL after bed rest. RVE (*n*=7) only significantly decreased SNO-protein levels in both SOL (Pre 75.3±0.75, End 62.25±1.7) and VL (Pre 144.52±3.8, End 122.32±4.1). C. NOS1 WB analysis of BST streptavidin column eluate vs. normal muscle lysates (Muscle lysate). In both SOL and VL, NOS1 immunoreactive bands with higher relative mobility are detectable (BST/Eluate) vs. a predicted NOS1 immunoreactive band obtained from total muscle lysates used as positive control.

A significant increase in the amount of biotin-labeled proteins, reflecting the relative SNO-protein levels in muscle biopsies was clearly documented after bed rest in both SOL and VL muscle biopsy samples (CTR SOL 18.5%; VL 19%) (Fig. 1A). However, from the two types of countermeasures only RVE was able to prevent such changes in both types of muscles (SOL−19%; VL−17%). In the RE group, no change in slow-type SOL (3.3%) and a significant increase in mixed-fast-type VL (+16%) was found, suggesting the presence of muscle-specific (fast vs. slow type) SNO-protein levels (Fig. 1A). Control experiments performed by omitting the biotin-HPDP during the BST experimental protocol showed no biotin-labeled proteins in any bed rest samples, suggesting no interference of the endogenous biotin in our experiments (Fig. 1A Inset, line Control BST).

### S-nitrosylation of NOS1 (SNO-NOS1) protein in human skeletal muscle

**Fig. 2:**
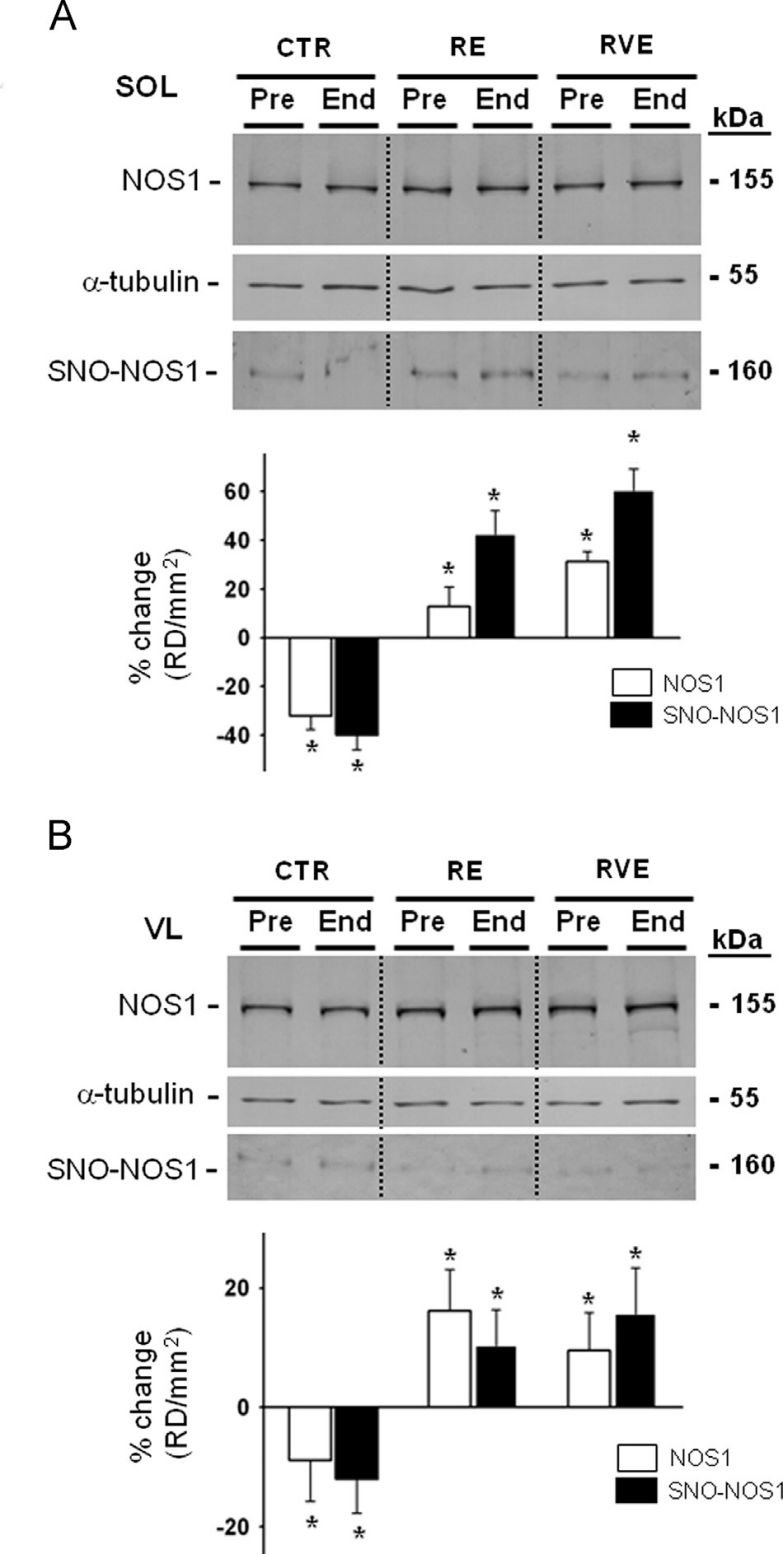
BST-related SNO-NOS1 protein assay of human SOL and VL muscle biopsies before and after bed rest. A. *Upper panel*, NOS1 WB analysis of SOL lysates normalized to alpha tubulin. A. Middle panel, NOS1 WB analysis of BST-streptavidin column eluate in SOL lysates. NOS1 protein immunoreactive bands reflecting biotin-labeled NOS1 proteins were present in all eluate samples showing the presence of S-nitrosylated NOS1 proteins in human SOL. A. *Lower panel*, graph representing percent changes of NOS1 (white columns, CTR (*n*=9), −32%, *p*≤0.01; RE (*n*=7), 12.7%, *p*≤0.05; RVE (*n*=7), 31.2%, *p*≤0.01) and of SNO-NOS1 (black columns, CTR (*n*=9), −40%, *p*≤0.01; RE (*n*=7), 42.5%, *p*≤0.01; RVE (*n*=7), 60.6%, *p*≤0.01) proteins in SOL of End vs. Pre bed rest biopsies (Pre values are set up as zero baselines). B. *Upper panel*, NOS1 WB analysis of VL lysates normalized to alpha tubulin. B. Middle panel, NOS1 WB analysis of BST-streptavidin column eluate in VL lysates. NOS1 protein immunoreactive bands reflecting biotin-labeled NOS1 proteins were present in all eluate samples showing the presence of S-nitrosylated NOS1 proteins in human VL. B. *Lower panel*, graph representing percent changes of NOS1 (white columns, CTR (*n*=9), −9.84%, *p*≤0.05; RE (*n*=7), 16.2%, *p*≤0.01; RVE (*n*=7), 9.5%, *p*≤0.05) and of SNO-NOS1 (black columns, CTR (*n*=9), −12%, *p*≤0.05; RE (*n*=7), 10.45%, *p*≤0.05; RVE (*n*=7), 15.65%, *p*≤0.01) proteins in VL lysates of End vs. Pre bed rest samples (Pre values are set up as zero baselines).

NOS1 is abundantly expressed in skeletal muscle and responsible for activity-dependent NO-signaling pathways in myofibers. As revealed by Western blot experiments, NOS1 protein was present in BST streptavidin column eluate (Fig. 1B) of both muscles. Note the shift in the relative mobility of the biotin-labeled NOS1 protein (SNO-NOS1, BST/Eluate) when compared to native NOS1 protein immunodetected in control (Muscle lysate) samples (Fig. 1b). As shown in Fig. 2A and B upper panel, NOS1 proteins were expressed in all Pre- and End-bed rest muscle biopsy lysates. Comparable levels of SNO-NOS1 proteins were present in both SOL and VL muscles biopsies of BST streptavidin column eluate of both Pre and End-bed rest subjects (Fig. 2A and B middle panel). A densitometry analysis confirmed a decrease of both NOS1 and SNO-NOS1 in the CTR group and an increase in the RE and RVE groups in both muscles after bed rest (Fig. 2A and B, lower panel).

### Anti-nitrosocystein (SNO-Cys) immunohistochemistry in human SOL and VL muscles

**Fig. 3:**
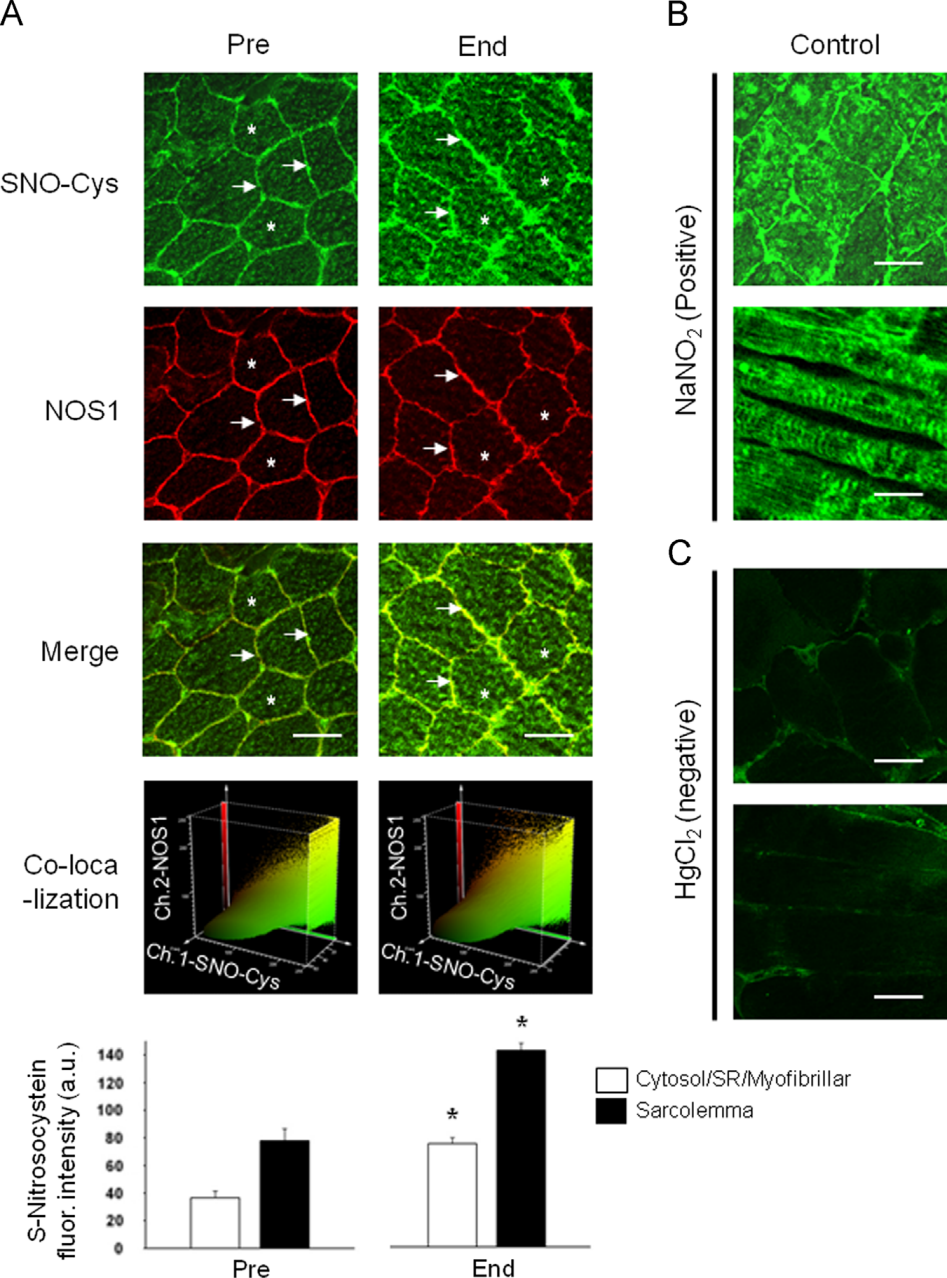
Confocal S-nitrosocystein (SNO-Cys) immunohistochemistry analysis in human skeletal muscle biopsies. A. Before start of bed rest (Pre) anti-SNO-Cys, anti-NOS1, and merged images (Merge). Anti-SNO-Cys antibodies immunolabelled the subsarcolemmal compartment (arrows, green fluorescence channel) that almost fully overlapped with anti-NOS1 antibodies immunostaining, red channel immunofluorescence signals (red and green merged yellow pixel area in 3D co-localization box plot at bottom panel), the cytosol/sarcoplasmic reticulum (SR)/myofibrillar compartment (asterisks). After bed rest (End), significantly increased SNO-Cys immunofluorescence was seen in SOL. A. *Lower panel*, graph representing anti-SNO-Cys immunosignal intensity in CTR Pre and End bed rest SOL. Significant changes in NOS1 (not shown) and SNO-Cys proteins were detected by intracellular pixel area intensity analysis (CTR (*n*=5) Pre, 36.55, *p*≤0.01; CTR (*n*=5) End, 75.55, *p*≤0.01) and sarcolemmal (CTR (*n*=5) Pre, 77.78, *p*≤0.01; CTR (*n*=5) End, 143.89, *p*≤0.01). B. Positive control. Cryosection was pre-incubated with NaNO_2_ (producing nitroso compounds) followed by SNO-Cys immunostaining. C. Negative control. Cryosection was blocked by pre-incubation with HgCl_2_ followed by anti-SNO-Cys immunostaining. Bar=50 µm. (For interpretation of the references to color in this figure legend, the reader is referred to the web version of this article.)

SNO-Cys immunohistochemistry performed on cross-cut cryosections revealed a general pattern of myofiber SNO-proteins that we considered as steady-state (baseline) before bed rest was commenced. As shown in Fig. 3A (Pre column, green channel), a moderate SNO-Cys immunoreactivity was present in Pre-bed rest muscle biopsies of CTR subjects at the three main subcellular compartments (arrows): subsarcolemmal, cytosolic, and sarcoplasmic reticulum-myofibrillar.

Confocal analysis of sarcolemma membrane areas confirmed an overlapping pattern of anti-SNO-Cys with anti-NOS1 immunosignals (Fig. 3A, Pre, green and red channels) that was considered as baseline co-immunolocalization of SNO-Cys and NOS1 at the sarcolemma region.

After bed rest significant increases in subsarcolemmal and intracellular SNO-Cys immunofluorescence were clearly observable in the CTR group (Fig. 3A, End). This could be additionally confirmed by confocal S-nitrosocystein fluorescence intensity analysis (Fig. 3A, lower panel), thus suggesting that prolonged muscle disuse in bed rest induced an augmented relative immunoexpression pattern of muscular SNO-proteins at various subcellular myofiber compartments (Fig. 3A) which was prevented by RE and RVE countermeasure protocols (data not shown). Positive control experiments using sodium nitrite showed a significant increase in anti-SNO-Cys fluorescence intensity within myofiber subcellular compartments (Fig. 3B, Control), confirming specificity of anti-SNO-Cys antibodies immunolabelling that was abolished by pre-incubation of the tissue with mercury chloride a blocking reagent solution (Fig. 3C, negative control).

### Fast- and slow-type SNO-myosin heavy chain (MyHC) proteins

**Fig. 4:**
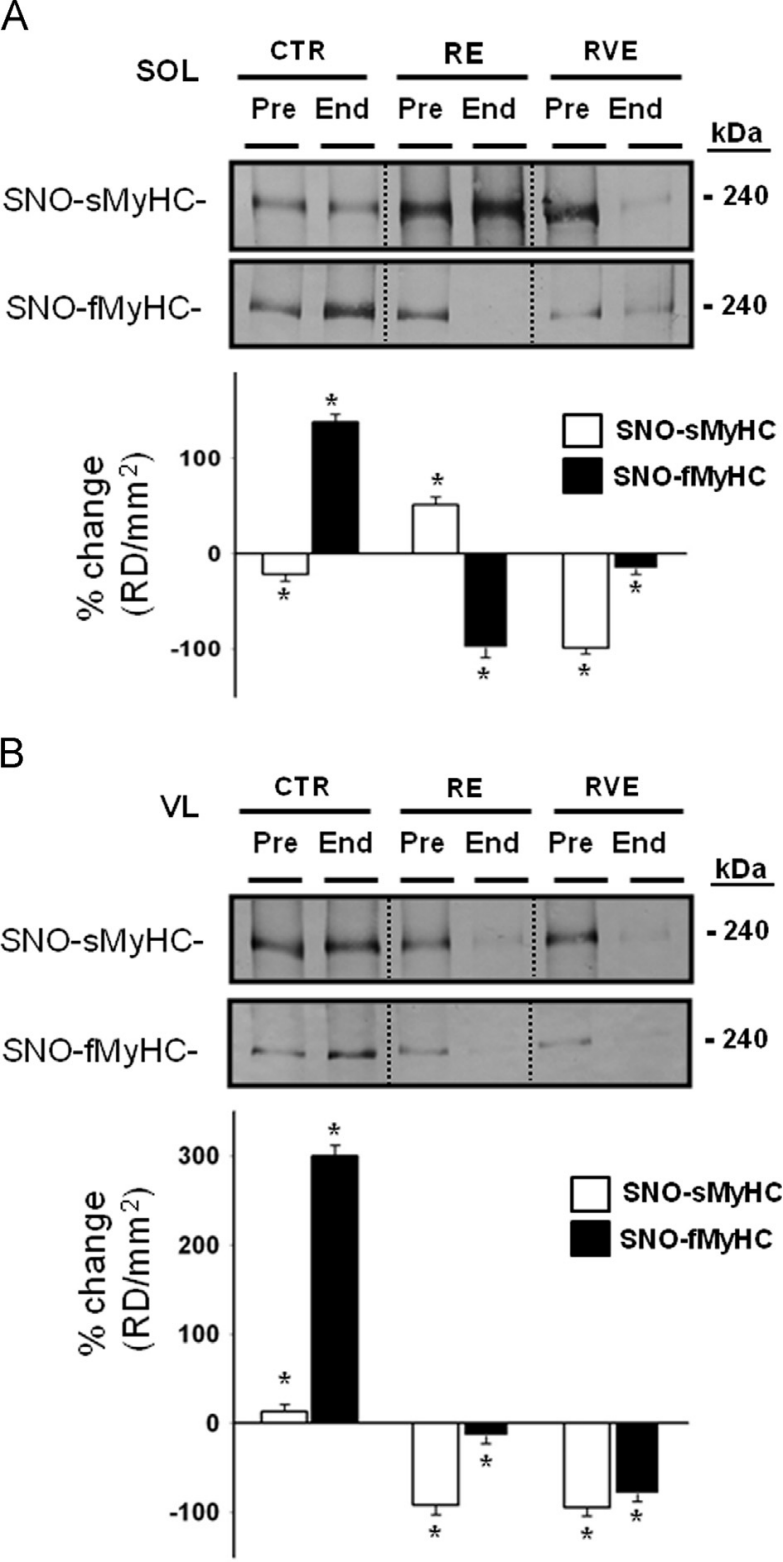
BST detection of SNO-myosin heavy chain (MyHC) proteins in bed rest muscle biopsies. A. Immunoblotted BST streptavidin column eluate of SOL investigated for the presence of fast- (*upper panel*) and slow-type (*lower panel*) MyHC. Significant decrease in slow-type (−22%, *p*≤0.05) and increase in fast-type (138%, *p*≤0.01) MyHC was present in CTR (*n*=9) End bed rest biopsies. In the RE group (*n*=7), an increase of slow-type (51%, *p*≤0.01) and a decrease of fast-type (−99%, *p*≤0.01) MyHC was seen. In the RVE (*n*=4) group, both slow-type (−99%, *p*≤0.01) and fast-type (−16%, *p*≤0.05) MyHCs levels were equally decreased. B. Slow- and fast-type MyHC (s/fMyHC) immunoblot analysis of BST streptavidin column eluate of VL. Significant increase of slow-type (13%, *p*≤0.05, *lower panel*) and fast-type (300%, *p*≤0.01) MyHCs were present in CTR (*n*=9) End bed rest biopsies. In the RE group (*n*=7), a decrease of slow-type (−92%, *p*≤0.01) and fast-type (−15%, *p*≤0.05) MyHC was present. In the RVE group (*n*=7), both slow- (−95%, *p*≤0.01) and fast-type (−80%, *p*≤0.01) MyHC levels were equally decreased.

MyHC proteins are abundantly expressed key proteins of the myofibrillar protein fraction that could well be targets for aberrant SNO modifications in disused muscle. A significant increase in biotin-labeled fast-MyHC proteins reflecting SNO-fast-MyHC proteins was present in SOL (138%) and VL (300%) of End-bed rest CTR group, while both RE and RVE countermeasure protocols prevented or even decreased (RE SOL, −99%, VL, −15%; RVE SOL, −16%, VL, −80%) such modifications in both muscles (Fig. 4A,B, upper panel). Conversely, a decrease in biotin-labeled slow-MyHC, was documented in SOL (−22%) but not in VL (13%) of End-bed rest CTR group. In RE, however, a significant increase in slow SNO-MyHC proteins was present in SOL (51%) that was not detectable in VL (−92%). Only RVE was able to prevent or decrease (SOL −99%; VL −95%) the levels of slow SNO-MyHC proteins in both SOL and VL muscles following bed rest (Fig. 4).

### SNO-RyR1

**Fig. 5:**
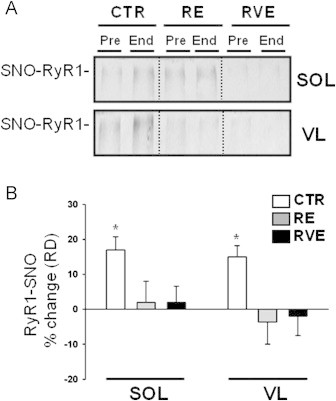
BST detection of SNO-RyR1 in human skeletal muscle SOL and VL biopsies. A. *Upper panel*, Immunoblot of BST streptavidin column eluate of SOL for RyR1. A. *Lower panel*, Immunoblot of BST streptavidin column eluate of VL for RyR1. Increased RyR1 immunoreactive band was detected in CTR End-only samples. B. Percent changes biotin-labeled RyR1 proteins from Pre and End bed rest biopsies. Bed rest without exercise promoted *S*-nitrosylation of RyR1 in disused SOL (CTR *n*=9, 18.6%, *p*≤0.01) and VL (CTR *n*=9, 17.2%, *p*≤0.01) as counteracted by both exercise countermeasures. (* significance).

The ryanodine receptor protein type 1 (RyR1) represents the major intracellular Ca^2+^-release channel responsible for calcium efflux from sarcoplasmic reticulum to the cytosolic compartment. We found moderate SNO-RyR1 protein levels in all Pre-bed rest samples reflecting baseline (Fig. 5A). However, in CTR without exercise (CTR End-bed rest), a significant increase in SNO-RyR1 protein levels was present in biopsies of both SOL (18.6%, Fig. 5B) and VL (17.2%, Fig. 5B). Conversely, both exercise countermeasure protocols (RE or RVE) counteracted such excess levels of RyR1 post-translational SNO modifications in both SOL and VL muscle (Fig. 5B).

It is well known that RyR1 channel activity is modulated by calcium binding protein calsequestrin (CSQ) 1 and 2. To further study RyR1 regulatory proteins, the presence of CSQ 1 and 2 was investigated in the BST streptavidin column eluate. CSQ 1 or 2 were never detectable in any of the Pre and End BR muscle samples (data not shown) suggesting that CSQ proteins could be used as a negative internal control biomarkers (lacking SNO-moieties) in our human skeletal muscle experiments.

### SNO-SERCA

**Fig. 6:**
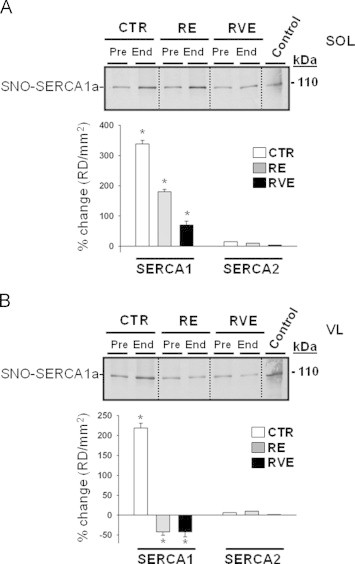
BST detection of sarcoplasmic reticulum SNO-SERCA1/–SERCA2 in human skeletal muscle SOL and VL biopsies from bed rest groups. A. *Upper panel*, Immunoblot of SERCA1 in BST streptavidin column eluate of SOL; *lower panel*, percent changes SNO-Cys-SERCA1 and SNO-Cys-SERCA2 of End vs. Pre bed rest SOL (Pre values are set up as zero baselines). An increase of SNO-SERCA1 in CTR (330%, *p*≤0.01, *n*=9), RE (180%, *p*≤0.01, *n*=7), and RVE (75%, *p*≤0.01, *n*=7) group was detected (*). No changes were observed for SNO-SERCA2 proteins. B. *Upper panel*, Immunoblot of SERCA1 of streptavidin column eluate of VL; *lower panel*, percent changes SNO-Cys-SERCA1 and SNO-Cys-SERCA2 of End vs. Pre bed rest VL (Pre values are set up as zero baselines). An increase of SERCA1 (220%, *p*≤0.01, *n*=9) was present in CTR End subjects, while a decrease was present in RE (-41%, *p*≤0.01, *n*=7) and RVE (-40%, *p*≤0.01, *n*=7) subjects (*). No changes were observed for SNO-SERCA2 proteins.

The sarco(endo)plasmic reticulum Ca^2+^-dependent ATPase SERCA1a is responsible for Ca^2+^ uptake from the cytosol into the sarcoplasmic reticulum lumen and intracellular calcium store of fast-type myofibers. Like RyR1, however, moderate SNO-SERCA levels were found in all Pre bed rest samples (Fig. 6A and B, upper panel). Following disuse in bed rest, a significant increase of SNO-SERCA1a levels was present in SOL (330%) or VL (220%) of CTR group (Fig. 6A and B, upper and lower panel). Thus, high amounts of SNO-SERCA1a protein levels were documented following extended muscle disuse independently of the muscle type. However, opposite results were obtained in the RE group (SOL, 180%; VL, −41%), suggesting that RE highly induced functional SNO-SERCA protein levels in a typically muscle-specific pattern. Similar results were found in the RVE group with the sole difference that in SOL an increase of the SNO-SERCA1 levels was more attenuated (75%), while in VL the SNO-SERCA1 levels were largely maintained or reduced (−40%, Fig. 6A and B, lower panel).

SNO-SERCA2a protein levels specifically expressed in slow-type myofibers (immunoblots not shown) by densitometry analysis (Fig. 6A and B, lower panel) were found unchanged after bed rest muscle disuse in all groups. These results confirm that a moderate SERCA1 and SERCA2 S-nitrosylation may be principally required for normal SERCA-ATPase activity in both muscles – independent of muscle activity, whereas only SERCA1 is specifically affected by nitrosative stress following extended muscle disuse.

### SNO-DHPR1α

**Fig. 7:**
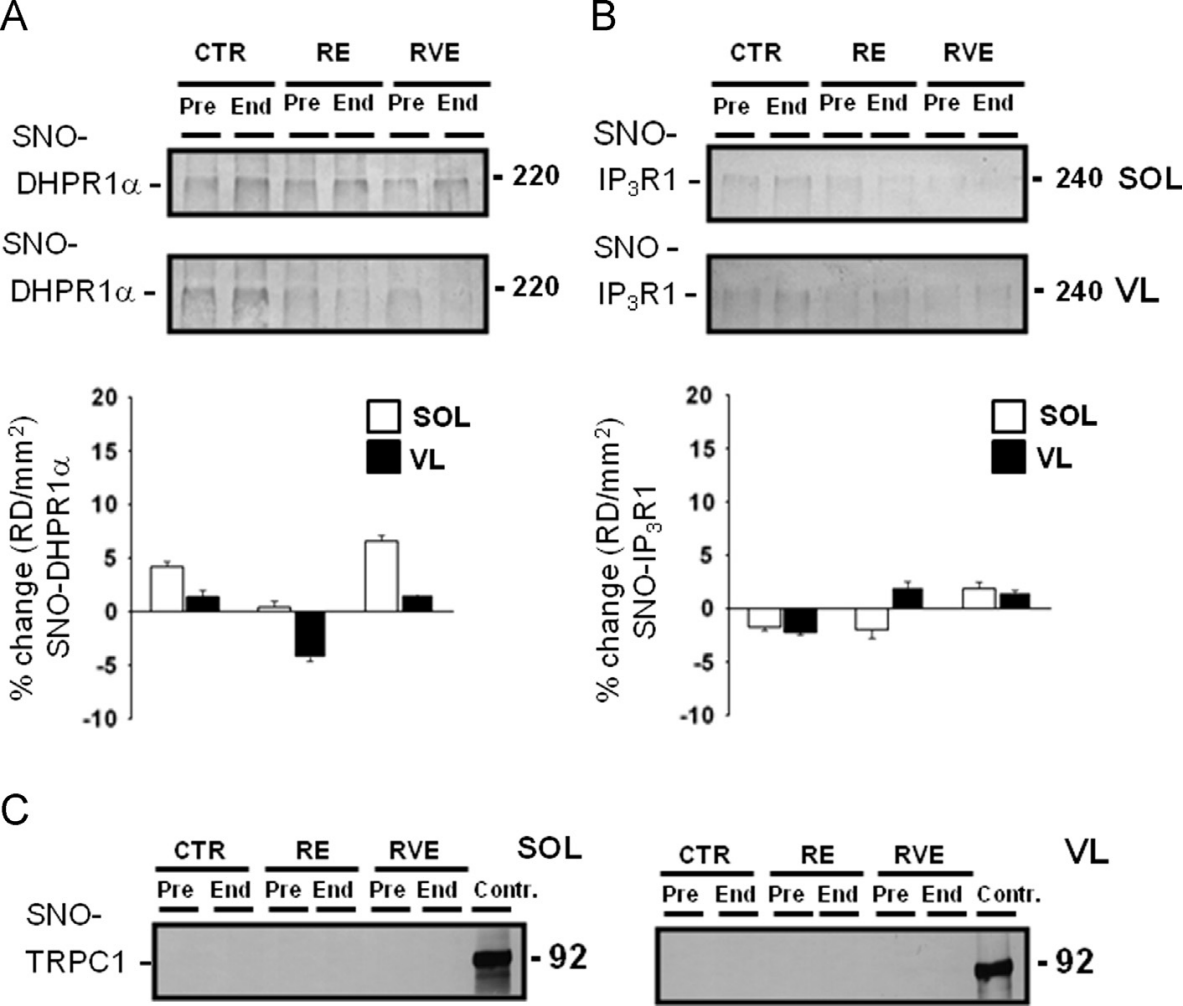
BST detection of sarcolemmal and sarcoplasmatic reticulum functional channel proteins in human skeletal SOL and VL from bed rest subjects. A. *Upper and lower panel*, DHPR1*α* immunoblot of BST streptavidin column eluate of SOL and VL. A moderate presence of DHPR1*α* proteins was present in all groups (CTR *n*=9; RE *n*=7; RVE *n*=7). B. *Upper and lower panel*, IP_3_R1 immunoblot of BST streptavidin column eluate of SOL and VL. A moderate presence of IP_3_R1 proteins was present in all groups (Pre). No changes were present in both samples after bed rest (End) (CTR *n*=9; RE *n*=7; RVE *n*=7). C. *Left and right panel*, TRPC1 immunoblot of BST streptavidin column eluate of SOL and VL (CTR *n*=9; RE *n*=7; RVE *n*=7); (Contr.=positive control of total proteins muscle lysates). No SNO-TRPC1 protein signals were detectable in either group before and after bed rest.

The voltage-gated ion channel dihydropiridine receptor 1 alpha (DHPR1α) is expressed at the T-tubule sarcolemma infolding of skeletal myofibers and is reported to be functionally linked (by physical contact) to the intracellular Ca^2+^ release channel RyR1. In our experiments, comparable levels of SNO-DHPR1α antigens were present in all bed rest BST/streptavidin column eluate (Fig. 7A, upper and middle panel) suggesting that a moderate level of S-nitrosylated DHPR1α is required for the physiological regulation of their functional activity. However, densitometry analysis revealed no significant changes in the levels of SNO-DHPR1α proteins were present in either SOL or VL muscle biopsies following bed rest only or bed rest with exercise countermeasures (Fig. 7A, *lower panel*).

### SNO-IP_3_R1

Inositol 1,4,5-trisphosphate receptor 1 (IP_3_R1), together with the RyR1, is one of the two well-characterized intracellular calcium release channels in skeletal muscle. IP_3_R1 antigens were present in all Pre and End bed rest of BST/streptavidin column eluate (Fig. 7B, upper and middle panel). However, by densitometry analysis, no significant changes in SNO-protein levels were present in both SOL and VL muscle biopsies after the bed rest period (Fig. 7B, lower panel) suggesting that – similarly to the DHPR1α results – moderate SNO-IP_3_R1 protein levels are required for a physiological regulation of their functional activity.

### SNO-TRPC1

The transient receptor potential (TRP) channel is a Ca^2+^ channel protein localized at the outer plasma membrane and responsible for calcium influx from extracellular compartment into intracellular myofiber compartment. TRPC1 antigens were not found in Pre and End bed rest muscle biopsies of BST/streptavidin column eluate of both SOL and VL muscle (Fig. 7C). A predicted TRPC1 immunoreactive band of 92 kDa was present on total muscle lysates used as positive control. Taken together these results suggested that no functional regulation mechanisms through S-nitrosylation mechanisms are present on TRPC1 in human SOL and VL skeletal muscle.

### SNO-PMCA1

**Fig. 8:**
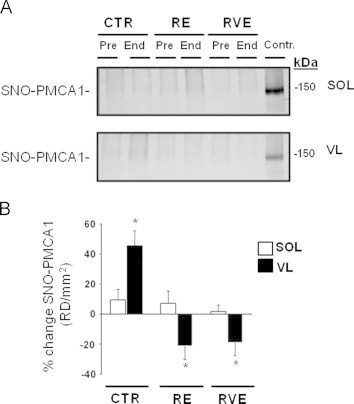
BST detection of sarcoplasmic membrane SNO-PMCA1 in human skeletal muscle SOL and VL from bed rest groups. A. *Upper panel*, PMCA1 immunoblot BST streptavidin column eluate of SOL (CTR *n*=9; RE *n*=7; RVE *n*=7); a faint PMCA1 immunoreactive band was present in all groups. A. *Lower panel*, PMCA1 immunoblot of streptavidin column eluate of VL (CTR *n*=9; RE *n*=7; RVE *n*=7); a faint PMCA1 immunoreactive band was present in all groups. Control=positive control with human endothelial cell lysates (BD Biosciences). B. Percent change SNO-PMCA1 in SOL and VL of all groups. No changes in SNO-PMCA1 were found SOL (CTR 9.4%, *p*>0.05; RE SOL 7.5%, *p*>0.05, RVE 1.78%, *p*>0.05) while changes were present in VL (CTR 45.55%, *p*≤0.01; RE −21.35%, *p*≤0.01; RVE −19.07%, *p*≤0.01) after bed rest (* significance).

The plasma membrane Ca^2+^-ATPase type-1 (PMCA1) is responsible for Ca^2+^ extrusion from the intracellular (cytosol) to the extracellular myofiber compartment. As revealed by anti-PMCA1 immunoblotting experiments, comparable levels of PMCA1 antigens were present in all Pre-bed rest streptavidin-column eluate of each group and muscle (Fig. 8A, upper and lower panel). These findings are consistent with our SERCA1 results, suggesting that moderate S-nitrosylation of PMCA1 (SNO-PMCA1) might be required for physiological regulation of *Ca*^2+^*-ATPase* activity in normal human skeletal muscle fibers (Fig. 8A). After bed rest, however, a trend to enhancement of SNO-PMCA1 levels was seen in SOL biopsies of CTR and RE End bed rest subjects while in VL biopsies there was a significant increase only in End bed rest biopsies of CTR subjects. Conversely, the RE and RVE countermeasure protocol significantly decreased SNO-PMCA1 in VL biopsies (Fig. 8B).

Due to the low relative protein expression of PMCA1 in skeletal muscle when compared to the robust levels of SERCAs, RyR1 and/or MyHCs, the SNO-PMCA1 protein analysis should be further investigated to verify our findings.

### Nuclear factor-erythroid 2-related factor 2 (Nrf-2) transcripts in SOL and VL muscle biopsies of BR subjects

**Fig. 9:**
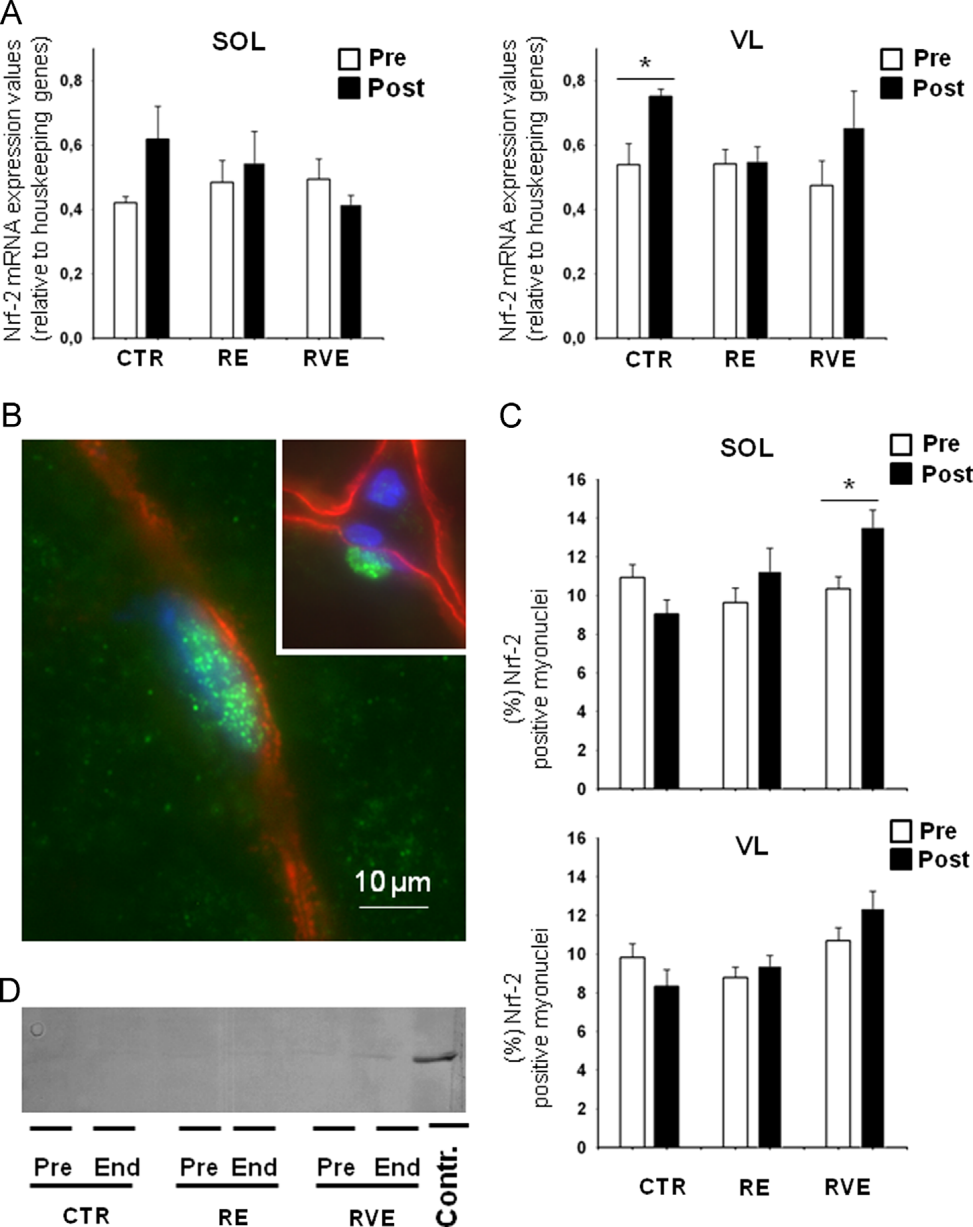
Anti-oxidative master gene Nrf-2 regulation in SOL and VL of bed rest subjects. A. Quantitative PCR analysis with Nrf-2 specific probe in SOL (left panel) and VL (right panel). Increase in Nrf-2 transcripts was detected after bed rest only in CTR group in VL (+39.33%, *P=*0.016*). B. Nrf-2 immunopositive myonucleus (green) in SOL after bed rest. Dystrophin (red) was used to label the subsarcolemmal border; nuclei were counterstained with DAPI (blue). Inset, positive Nrf-2 myonucleus for comparison with Nrf-2 negative nuclei outside the myofiber. C. Quantitative analysis of Nrf-2 positive myonuclei in SOL (upper panel) and VL (lower panel). The total number of Nrf-2 positive myonuclei was slightly reduced in both, SOL (Pre, 10.93±0.66%, End, 9.03±0.75%) and VL (Pre, 9.83±0.71%, End, 8.33±0.86%) of CTR group after bed rest. In the RE group no changes were seen in SOL (Pre, 9.62±0.77%, End, 11.25±1.19%) and VL (Pre, 8.77±0.54%, End, 9.39±0.54%). In RVE group a increased numbers of Nrf-2 positive myonuclei were present in SOL (+30.55%, *P=*0.012*) but absent in VL (+17%, *P*=*0.15*). D. Nrf-2 immunoblot of VL muscle lysates in all groups. A faint Nrf-2 immunoreactive band with an apparent MW similar to the positive control (contr.) was detected in all samples. Contr.: Jurkat whole cell lysates (SC-2204, Santa Cruz Biotechnology, CA). (For interpretation of the references to color in this figure legend, the reader is referred to the web version of this article).

Nrf-2 transcripts were present in biopsies of SOL (left panel) and VL (right panel) from all pre-bed rest volunteers, although with slight differences in their relative expression levels (Fig. 9A). An increase in Nrf-2 transcripts after BR-only was detected in VL (Pre, 0.539±0.065; End, 0.751±0.023) whereas in SOL (Pre, 0.421±0.018; End, 0.618±0.1) a clear trend was found. However, no significant changes in Nrf-2 transcripts were detected in both muscles of either exercise countermeasure groups (Fig. 9A).

### Nrf-2 myonuclei translocation

A detailed morphometric analysis of Nrf-2 cytosol-to-myonuclei shuttling by triple-immunohistochemistry of biopsy cryosections was performed in all groups (Fig. 9B). Quantitative determination (Fig. 9C) showed that the percentage of Nrf-2 positive myonuclei was slightly reduced in both SOL (Pre, 10.93±0.66%, End, 9.03±0.75%) and VL (Pre, 9.83±0.71%, End, 8.33±0.86%) of CTR group after bed rest. In the RE group, a trend was seen in both SOL (Pre, 9.62±0.77%, End, 11.25±1.19%) and VL (Pre, 8.77±0.54%, Post, 9.39±0.54%). In the RVE group, a significant increase in Nrf-2 positive myonuclei was present in SOL (Pre, 10.35±0.62%, End, 13.53±0.89%), but not in VL (Pre, 10.68±0.68%, End, 12.36±0.88%). Although the percentage of Nrf-2 positive myonuclei was not always significantly changed in both muscles of all groups, a clear trend between groups without and with exercise countermeasures was present. Thus, exercise intervention in bed rest (RE, RVE) is apparently reflected by altered patterns of Nrf-2 nucleocytoplasmic shuttling in either human skeletal muscle, preferentially in SOL after 60 days of bed rest.

By Western blot analysis a faint Nrf-2 immunoreactive band was detected in all bed rest biopsies RIPA extracts (Fig. 9D). However, since part of the protein amount could be retained in the pellet nuclei, further studies are required to define the cytosolic and nuclei fraction ratio. Jurkat whole cell lysates were used as positive control.

## Discussion

We propose here that altered protein S-nitrosylation likely reflects variable nitrosative stress management affecting components of the excitation–contraction (EC) coupling machinery, and thus calcium homeostasis in disused human skeletal muscle following 60 days of bed rest.

Protein S-nitrosylation of functional muscle proteins most likely serve as a key mechanism in the routine control of intracellular calcium homeostasis in active skeletal muscle [18,19] while aberrant SNO posttranslational modifications such as in inactive/disused muscle may account for leaky calcium channels due to increased nitrosative stress, as previously proposed for atrophic muscle fibers [2,20,21] or by exposure to redox modifying agents [22].

Our results support the hypothesis that disuse-induced muscle atrophy results from disrupted Ca^2+^ homeostasis, i.e., substantial increase of cytosolic free Ca^2+^ concentration as previously proposed [23,24] which in turn may cause activation of several Ca^2+^-dependent proteolysis/signaling pathways [25,26].

Thus, we here speculate that excess levels of SNO-proteins are likely to promote disuse-induced atrophy via calcium/calpain-associated proteolysis in human skeletal muscle during bed rest. Evidence supporting this hypothesis is provided by the increased biotin labeled (reflecting S-nitrosylated) levels of intracellular Ca^2+^-release and Ca^2+^-uptake proteins such as the sarcoplasmic reticulum-associated RyR1 and SERCA1 and, most probably, the plasma membrane-associated PMCA1, or even the slow and fast MyHC proteins of the myofibrillar contractile apparatus suggestive for altered individual components of the E–C coupling machinery. Accordingly, this would be the first study showing elevated S-nitrosylation levels of specific E–C coupling regulatory and contractile proteins that are inversely regulated by human muscle activity.

The present results are in agreement with previous studies showing increased protein oxidative stress by muscle protein S-nitrosylation [16,27] and/or carbonylation [28,29], or even altered total thiol protein (SH) group concentrations and antioxidant activity [30] after bed rest, or S-nitrosylation of MyHCs in rat skeletal muscle [31].

Altered S-nitrosylated proteins detected in the present study may, at least partially, be due to changes in the relative protein expression and changes in the intrinsic protein S-nitrosylation ratio, for example, in response to altered muscle activity [12,32] or myofiber phenotype transition [33]. Nevertheless, the observed changes in protein S-nitrosylation ratio of MyHCs in the RE /RVE exercise groups clearly do not reflect major changes in the relative protein expression (Fig. 4A and B). In addition, the relatively low protein expression of some functional proteins (like PMCA) in our protein preparations or the reduced protein mobility because of biotin incorporation levels (like RyR1, a tetramer subunits channel of approx. 565 kDa with at least 50 Cystein groups each), pull down assays and subsequent electrophoresis may not always result in sharp protein bands thus showing some limitations of the modified BST method used in the present work.

The amount and duration of S-nitrosylation of muscle-specific proteins are thought to exert either inhibitory (negative) or activating (positive) effects following nitrosative stress conditions in various models [2]. While moderate SNO-patterns may be required for normal activation of e.g. SERCA muscle protein [19], elevated SNO-patterns may account for inhibitory effects [2], thus suggesting that SNO regulatory functions in physiological and/or patho-physiological conditions are even more complex.

The present findings are based partly on indirect biochemical SNO-protein detection of total muscle proteins based on BST [7], combined with immunoblot mapping of key proteins related to the control of calcium homeostasis. SNO-Cys immunohistochemistry at subcellular myofiber compartments typically revealed muscle-specific distribution patterns of putative SNO-proteins that were attributable to both muscle phenotype (slow vs. fast type) or possibly the mode of physical exercise (RE vs. RVE) performed as countermeasure in bed rest. Nevertheless, even if the BST method has some limitations [34], we were able to perform a functional SNO-protein screening showing variable nitrosative stress management in disused vs. trained human skeletal muscle. The SNO-protein analysis performed here by indirect detection via BST neither discriminates between hypo- or hyper-S-nitrosylation of individually target proteins nor identifies exactly the specific SNO-sites in a given target protein [35].

The BBR2-2 Study protocol included two different countermeasure protocols, resistive exercise (RE) and resistive exercise combined with whole-body vibration exercise (RVE). This allowed for direct comparison of nitrosative stress management between the two modes of countermeasure exercise. Moreover, the present countermeasure protocols likely affected the activity of Nrf-2 master gene which in turn may regulate the transcriptional activity of anti-oxidant response elements (AREs) of many cytoprotective genes [36]. Even if the outcome of both exercise regimens in bed rest was not fully complementary, we propose that both RVE and RE should generally be considered as effective countermeasure protocols to mitigate nitrosative stress conditions in skeletal muscle following extended strain conditions such as in bed rest immobilization.

Due to the particular nature of nitroso groups (relative short half-life and their reactivity in the cell) the question arises whether or not changes in nitrosation may have occurred earlier in bed rest. Short and/or a midterm term bed rest studies are required in the future to study the time course of protein nitrosation in muscle disuse.

Strenuous endurance exercise was reported to increase [37] rather than to decrease nitrosative stress. However, the present findings indicate that short bouts of high-load resistive exercise performed at regular intervals (3×5 min. each/2× week) during 60 days of bed rest mitigated high levels of disuse-induced nitrosative stress in muscle found in the bed-rest-only group. This may be explained by the two different muscle activity models i.e. exercise countermeasure following body unloading in healthy subjects in bed rest vs. normal and/or endurance muscle activity performed in healthy sport athletes [37]. Nitrosative stress management therefore seems to be a critical determinant for both acute overload in muscle exercise in sports (i.e., leading to fatigue or soreness) and chronically unloaded skeletal muscle in bed rest (i.e. disuse strain/atrophy mechanism) that appears to be attenuated by exercise as countermeasure.

NO-signaling plays a pleiotropic role in skeletal muscle biology [8]. For instance muscular NOS isoforms are activity-dependently expressed [38–40]. Nevertheless, experimental evidence for altered SNO-modifications of NOS proteins themselves are less well documented [41]. We here proposed NOS1 as a new candidate on the list of major functional SNO-proteins in human skeletal muscle. This unexpected new finding supports the notion that, in addition to both calcium sensitivity [8] and co-expression of specific regulatory proteins, the muscular NOS enzyme activity might be self-regulated via endogenously released NO-signals, thereby controlling the level of intracellular NO formation and bioactivity in a given skeletal myofiber. These observations are in agreement with the proposed presence of a SNO-NOS1 complex analyzed in a recombinant protein in vitro model [42–44]. Regulation of NOS activity can be also modified by phosphorylation mechanisms [45] and in response to myoplasmic calcium fluctuations [46], while NOS isoforms were identified only recently as endogenous NO primary sources [47]. Our present findings therefore support the notion that NOS1 can be auto-S-nitrosylated by local NO-signals in muscle fibers. This would in turn suggest the presence of an additional yet poorly defined auto-regulatory component of muscular NOS1 proteins as an additional regulatory paradigm in skeletal muscle physiology/pathophysiology in view of the recently proposed S-nitrosylases and/or denitrosylases as intracellular regulators [35]. To further confirm such NOS1 auto-regulation via SNO signaling, however, additional experiments using pharmacological approaches with NO-donors and/or NO-blocking agents using relevant in vitro or animal models are necessary and should complement the data from the present human study.

Some of the functional muscle proteins investigated in this study, including their putative SNO-sites and potentially SNO-substrates, have only recently been listed using theoretical computational prediction methods [48] or have been otherwise reviewed [35]. Alternative SNO-proteome approaches are clearly needed to further show if altered SNO-protein levels found in inactive vs. active skeletal muscle in bed rest are due to decreased or increased S-nitrosylation of specific SNO-sites on individual functional SNO-proteins such as the calcium release and calcium uptake channels as sketched in the graphical abstract (Online version). Nevertheless, we present first experimental proof for both the presence and the regulation of defined functional SNO-proteins related to calcium homeostasis in human skeletal muscle following variable nitrosative stress conditions via posttranslational SNO-protein modifications in normal vs. disused/atrophic skeletal muscle.

## Conclusions

The present report provides multiple strands of evidence for altered patterns of SNO-proteins including NOS1 itself as signatures of a unique nitrosative stress management in atrophic vs. trained skeletal muscle following bed rest immobilisation in slow or mixed-fast human muscle types. Due to the complexity of the molecular mechanisms and the functional consequences for individual muscle cell signaling pathways further investigation of SNO-protein dysregulation is mandatory. Determination of functional SNO-protein levels in biopsies of the two exercise bed rest groups provided a thorough basis for re-assessment of efficient exercise countermeasure protocols. Both RVE, and to lesser extent RE, significantly reduced disuse-induced aberrant SNO-protein levels and mitigated anti-oxidative master gene over-expression following bed rest. Nitrosative stress conditions are therefore instrumental in the regulation of skeletal muscle atrophy or maladaptation following whole body immobilisation that can be offset by exercise as countermeasure in various clinical settings, in rehabilitation, and during microgravity exposure in human spaceflight.

## Role of the funding source

The role of the funding sources was financial contribution/support. The sponsors did not participate in study design, collection, analysis and interpretation of data, writing of the report, or in the decision to submit the article for publication.
